# Utilization of standardized patients and case studies to evaluate effect of SBIRT training for APRN's

**DOI:** 10.1186/1940-0640-10-S2-O31

**Published:** 2015-09-24

**Authors:** Deborah MacMillan, Sheryl Winn, Sallie Coke, Annie Biers, Sylvia Shellenberger

**Affiliations:** 1 School of Nursing, Georgia College & State University, Milledgeville, Georgia 31061, USA; 2 School of Medicine, Mercer University, Macon, Georgia 31206, USA

## Background

Research supports the efficacy of screening, brief intervention, and referral to treatment (SBIRT) in reducing unhealthy substance use, and demonstrates that nurses are effective SBIRT providers. The purpose of utilizing MI is to help clients to manage their health independently, while building their confidence and willingness to change behavior[1]. Identifying effective pedagogical methods for teaching and evaluating students' proficiency in utilizing SBIRT is essential.

## Methods

Family Nurse Practitioner (FNP) students (n = 36) received SBIRT and Motivational Interviewing (MI) didactic content and clinical practice during three consecutive semesters. The students' skills were assessed during the 1^st^ semester (Time One) and the 3^rd^ semester (Time Two) using standardized patient case scenarios. Student encounters were recorded and evaluated by faculty, standardized patients and self-reflections. Debriefing sessions provided face to face feedback by faculty and MI trainers.

## Results

The percentage of students who were able to demonstrate six specific SBIRT skills increased from Time One measure to Time Two measure in four areas: identifying risk (82.6% vs.100%), educating on low risk drinking limits (67.4% vs. 97.2%), recommendation to quit or cut back (78.3% vs. 97.2%), and enhancing motivation (67.4% vs. 77.8%). The remaining two areas showed a slight decrease in the percentage of students who demonstrated those skills: asking permission to raise the subject (97.8% to 94.4%) and explaining the connection between substance use and the reason(s) for patient's current medical visit (89.1% vs. 88.9%). Student's self-reflections accurately identified areas of strengths and weakness in their individual SBIRT skills and were consistent with faculty evaluations of their performance.

## Conclusion

The use of standardized patient case scenarios was shown to be an effective venue for faculty to evaluate student proficiency at performing SBIRT. Educating the FNP student in SBIRT and MI will result in more clients being screened and treated for alcohol or drug misuse and to empower these clients to independently manage their health with success.
